# Detection of Pseudorabies Virus in Hunting Dogs in Greece: The Role of Wild Boars in Virus Transmission

**DOI:** 10.3390/pathogens14090905

**Published:** 2025-09-09

**Authors:** Konstantinos Papageorgiou, Ilias Bouzalas, Kiriaki Giamoustari, Małgorzata Wróbel, Dimitrios Doukas, Aikaterini Stoikou, Zoi Athanasakopoulou, Dimitrios Chatzopoulos, Dimitrios Papadopoulos, Spyridon Pakos, Chrysanthi Karapetsiou, Charalambos Billinis, Evanthia Petridou, Spyridon K. Kritas

**Affiliations:** 1 Laboratory of Microbiology and Infectious Diseases, School of Veterinary Medicine, Faculty of Health Sciences, Aristotle University of Thessaloniki, 54124 Thessaloniki, Greece; kiriakigi@gmail.com (K.G.); kstoikou@gmail.com (A.S.); dpapvet@hotmail.com (D.P.); epetri@vet.auth.gr (E.P.); skritas@vet.auth.gr (S.K.K.); 2 Veterinary Research Institute, Hellenic Agricultural Organization DIMITRA (ELGO-DIMITRA), Campus Thermi, 57001 Thessaloniki, Greece; bouzalas@elgo.gr; 3 Department of Microbiology and Clinical Immunology, Faculty of Veterinary Medicine, University of Warmia and Mazury in Olsztyn, Oczapowskiego Street 13, 10-719 Olsztyn, Poland; malgorzata.wrobel@uwm.edu.pl; 4 Laboratory of Animal Pathology and Veterinary Forensics, Faculty of Veterinary Medicine, University of Thessaly, 43100 Karditsa, Greece; ddoukas@vet.uth.gr; 5 Laboratory of Microbiology and Parasitology, Faculty of Veterinary Medicine, University of Thessaly, 43100 Karditsa, Greece; zathanas@uth.gr (Z.A.); billinis@uth.gr (C.B.); 6 Laboratory of One Health, Infectious Diseases and Zoonoses, Department of Public and One Health, University of Thessaly, 43100 Karditsa, Greece; dchatzopoulos@uth.gr; 7 Veterinarian, 45500 Ioannina, Greece; spyrospakos@gmail.com; 8 Veterinary Directorate, Region of Epiros, 45332 Ioannina, Greece; ch.karapetsiou@php.gov.gr

**Keywords:** wild boar, pseudorabies, dog, encephalitis

## Abstract

Aujeszky’s disease, or pseudorabies, is a viral infection caused by *Suid herpesvirus 1* (pseudorabies virus), with swine as its natural host. Although eradicated in domestic pigs in many European countries, PRV remains endemic in wild boar populations, posing a risk to other species, including carnivores. In this study, we report eight fatal cases of PRV infection in hunting dogs from Epirus and Thessaly, Greece, all of which followed direct contact with hunted wild boars. Postmortem brain samples tested positive for PRV via PCR targeting the *glycoprotein C (gC) gene*. Partial sequencing and phylogenetic analysis of the amplified gC fragments revealed genetic divergence among the examined isolates. The Epirus-derived strains formed a distinct cluster, closely related to previously reported Greek strains from the region of Central Macedonia as well as to the French strain FRA 527 and the German isolate GER614BW. In contrast, the two Thessaly sequenced isolates were phylogenetically distant from all other Greek strains, potentially representing an independently evolving lineage, and clustered more closely with the Kaplan strain. These findings underscore the persistent threat of PRV transmission from wild to domestic species and highlight the genetic heterogeneity of PRV strains circulating in Greece. Veterinary practitioners should consider PRV in the differential diagnosis of encephalitic symptoms in hunting dogs. Enhanced molecular surveillance and public awareness are critical to mitigating the risks posed by this emerging threat.

## 1. Introduction

Aujeszky’s disease, also known as pseudorabies, is an economically important disease of swine, occasionally affecting other domestic and wild animal species [1,2]. Pseudorabies is caused by *Suid alphaherpesvirus 1* (SuHV-1), classified within the genus *Varicellovirus*, subfamily *Alphaherpesvirinae*, and family *Herpesviridae* [3]. SuHV-1, or pseudorabies virus (PRV), consists of an enveloped nucleocapsid containing a double-stranded DNA genome of approximately 145 kb that encodes 70 different proteins [4].

The disease was initially described in 1813 in cattle exhibiting severe pruritus and was termed “mad itch”. Aladar Aujeszky, the Hungarian veterinarian after whom the disease was named, first described, and reproduced it in 1902, demonstrating that the etiological agent was a virus and not a bacterium [5].

Domestic swine and wild boars are considered the natural hosts of PRV and may act as sources of infection for a wide range of animals, including carnivores, ruminants, and rodents [6,7]. The clinical signs of pseudorabies in pigs primarily depend on the virulence of the infecting strain and the age of the affected animal. In non-immune piglets, especially neonates, the disease is typically fatal and involves central nervous system symptoms. Weaners (3–9 weeks of age) exhibit similar but generally less severe signs. In older pigs, pseudorabies manifests mainly as respiratory disease in fatteners, abortion in sows, and reversible infertility in boars [6,8,9].

Although pseudorabies has been eradicated from domestic pigs in many European countries—including Austria, Cyprus, the Czech Republic, Denmark, Finland, France, Germany, Hungary, Luxembourg, the Netherlands, Sweden, Switzerland, Norway, and the United Kingdom—it remains endemic in parts of Eastern and Southeastern Europe [2,10,11]. Successful eradication programs have relied on large-scale vaccination using gE-deleted vaccines, forming the basis of the DIVA (Differentiating Infected from Vaccinated Animals) strategy [12,13].

Despite its eradication in domestic pigs in many countries, PRV continues to circulate in wild boar populations [14,15,16,17,18,19]. Thus, wild boars play a pivotal role in the persistence of the infection in Europe and should be considered sources of PRV infection for domestic pigs and other wildlife species [20].

Carnivores are particularly susceptible to PRV infection, typically acquiring it through contact with infected wild boars [21]. Dogs, especially hunting dogs, become infected primarily through ingestion of uncooked offal from infected feral swine, although respiratory transmission has also been reported [22,23,24,25]. Clinical cases of PRV in dogs have been documented worldwide, particularly in regions where the virus circulates in domestic pigs and wild boars [26,27,28,29,30]. In dogs, pseudorabies is characterized by severe neurological signs caused by acute, fatal encephalomyelitis. Common clinical symptoms include dyspnea (60%), vomiting (36%), ataxia (76%), and muscle spasms (36%) [31]. Death typically occurs within 1–2 days after the onset of intense, localized pruritus of the head [21,27].

Greece is among the European countries where pseudorabies remains endemic. In a previous study, Papageorgiou et al. [32] reported that 28.6% of Greek pig farms tested positive for PRV antibodies. Farm selection was based on geographic criteria to ensure national representativeness. Furthermore, studies from 2015 [33,34] found PRV antibody prevalence ranging from 32% to 35% in wild boar populations. In another study, PRV DNA was detected in 17.3% of lung samples from hunted wild boars in Northwestern Greece [35].

The objective of this study was to characterize outbreaks of PRV infection in eight hunting dogs from Central and Northwestern Greece following contact with hunted wild boars, and to genetically characterize PRV isolates using phylogenetic analysis based on partial *gC gene* sequences.

## 2. Materials and Methods

### 2.1. Cases Presentation

**Figure 1:**
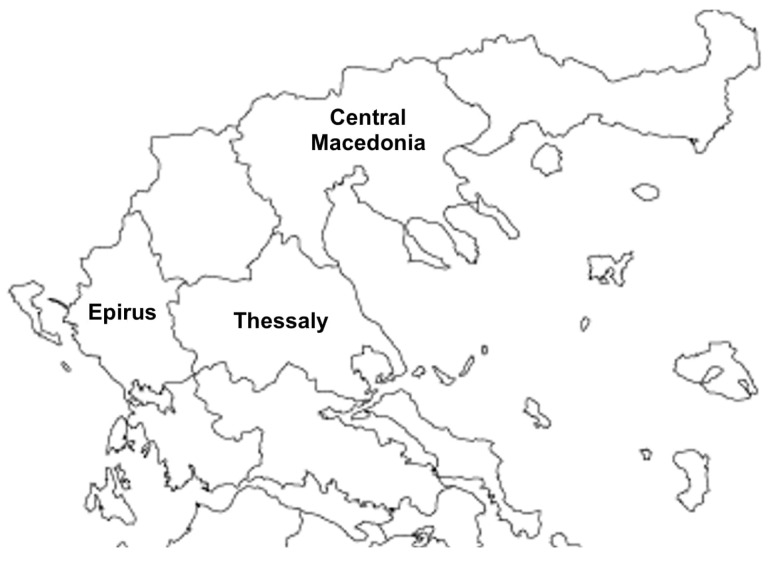
The Greek regions (Epirus–Thessaly) where the hunting dogs exhibited neurological and behavioral signs. In the region of Central Macedonia previously reported PRV strains Hercules and Kolchis had been isolated.

Between October 2021 and November 2024, eight hunting dogs from two distinct regions of Greece—Epirus (Northwestern) and Thessaly (Central) (Figure 1)—were referred to local veterinary clinics after showing neurological and behavioral signs.

Briefly, in October 2021, two dogs—a 2-year-old female and a 3-year-old male (designated dog 1 and dog 2)—were examined at a small animal veterinary clinic in the Epirus region. Clinical signs included severe pruritus localized to the head, tetraparesis, generalized muscle spasms, sialorrhea, and dyspnea. According to the owner, both dogs had participated in wild boar hunting a few days prior and had bitten into the carcass of a shot wild boar. Despite supportive care, no improvement was recorded and both animals died within 24–48 h after symptom onset. Following postmortem examination brain samples were collected from both dogs. The dog specimens were submitted to the Veterinary Research Institute, Hellenic Agricultural Organization—DIMITRA (VRI, ELGO-DIMITRA) for diagnostic testing. Samples were transported on the same day in isothermal containers maintained at approximately −20 °C.

In December 2021, another 2-year-old crossbreed female dog (dog 3) presented with neurological symptoms including paresis, hypersalivation, and intense pruritus of the head, five days after participating in wild boar hunting in Epirus. Given the clinical presentation and recent exposure history, PRV infection was suspected. The dog was euthanized due to the severity of symptoms, and its brain was submitted to VRI, ELGO-DIMITRA under the same conditions described above.

In October 2024, two additional 4-year-old crossbreed hunting dogs (dog 4 and dog 5) were euthanized after exhibiting characteristic signs of pseudorabies. Both had participated in wild boar hunting in Epirus and had consumed raw offal from the hunted animals. Postmortem brain samples were submitted to local authorities (Veterinary Directorate of the Epirus Region) and subsequently to the Laboratory of Microbiology and Infectious Diseases, School of Veterinary Medicine, Aristotle University of Thessaloniki, for virological examination.

**Figure 2:**
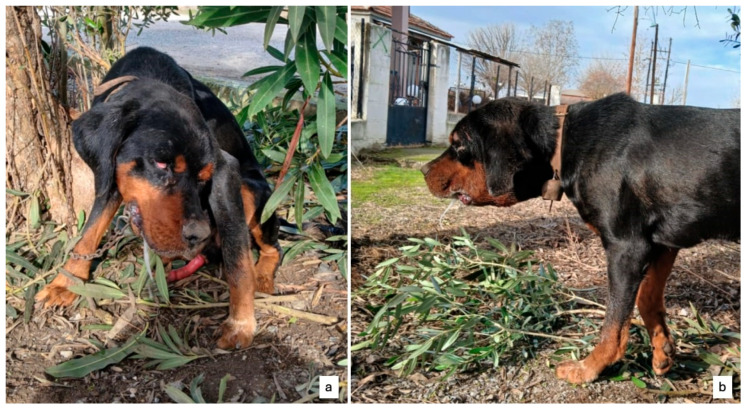
One of the three dogs (dog 6) from the Thessaly region reported with neurological signs. (**a**) Note the sialorrhea, characteristic head turning, and priapism. (**b**) The dog exhibiting pronounced sialorrhea.

Finally, in November 2024, veterinary assistance was sought for three crossbreed hunting dogs—aged 2, 3, and 4 years (dogs 6, 7, and 8, respectively)—in the region of Thessaly. All exhibited clinical signs of encephalitis, with symptoms including extreme pruritus of the head and sialorrhea (Figure 2). The owner reported recent participation in wild boar hunting in Thessaly and suspected ingestion of raw meat and offal from hunted animals. Due to the severity of their clinical condition, all three dogs were euthanized. On each dog, a full necropsy was performed at the Necropsy Hall of the Laboratory of Animal Pathology and Veterinary Forensics, Faculty of Veterinary Medicine, School of Health Sciences, University of Thessaly, without finding any specific gross lesion. Brain tissues were collected postmortem. One half of the brain was submitted to the Laboratory of Microbiology and Infectious Diseases, Aristotle University of Thessaloniki. As with previous cases, samples were transported in isothermal containers at approximately −20 °C. The other half of the brain was fixed in 10% neutral formalin fixative (Sigma-Aldrich, St. Louis, MO, USA) and processed routinely for histopathological examination according to Hematoxylin-Eosin (H-E) stain at the Laboratory of Animal Pathology and Veterinary Forensics, Faculty of Veterinary Medicine, School of Health Sciences, University of Thessaly.

All dogs had been vaccinated against rabies, and the diagnostic workup in all cases focused on the molecular confirmation of PRV as the etiological agent.

### 2.2. Polymerase Chain Reaction (PCR) and Sequencing

Genomic DNA was extracted from the brain tissue samples of every dog using commercially available DNA purification kits, following the manufacturers’ protocols. Specifically, the NucleoSpin^®^ Tissue Kit (Macherey-Nagel, Düren, Germany) was used for DNA extraction from samples submitted to the VRI, ELGO-DIMITRA (dogs 1–3), while the PureLink™ Genomic DNA Mini Kit (Invitrogen, Thermo Fisher Scientific Inc., Waltham, MA, USA) was employed for samples received at the Laboratory of Microbiology and Infectious Diseases, School of Veterinary Medicine, Aristotle University of Thessaloniki (dogs 4–8). The concentration and purity of the extracted DNA were assessed using a BioSpectrophotometer^®^ (Eppendorf, Vienna, Austria).

The presence of PRV DNA was investigated via PCR targeting the *gC gene*, as previously described by Muller et al. [30].

For samples from dogs 1–3, PCRs were carried out using the OneTaq^®^ 2X Master Mix with Standard Buffer (New England Biolabs, Ipswich, MA, USA). Each 25 μL reaction contained 12.5 μL of the 2Χ master mix, 0.5 μL of each primer (10 μM), 5 μL of template DNA, and nuclease-free water to a final volume of 25 μL. Amplification was performed in an ABI Veriti™ 96-Well Thermal Cycler (Applied Biosystems, Foster City, CA, USA) under the following cycling conditions: initial denaturation at 95 °C for 3 min; 35 cycles of denaturation at 94 °C for 15 s, annealing at 59 °C for 1 min and extension at 68 °C for 2 min; and a final extension at 72 °C for 4 min.

PCRs for samples from dogs 4–8 were conducted using the KAPA Taq HotStart PCR Kit (Sigma-Aldrich, St. Louis, MO, USA). Each 25 μL reaction mixture included 5 μL of 5Χ reaction buffer, 1.5 μL of MgCl_2_, 0.5 μL of a 10 mM dNTP mix, 1 μL of each primer (10 μM), 0.1 μL of Taq polymerase (5 U/μL), 5 μL of template DNA, and nuclease-free water up to 25 μL. PCR was performed using the same thermocycler model under the following conditions: initial denaturation at 95 °C for 3 min; 35 cycles of denaturation at 95 °C for 30 s, annealing at 59 °C for 1 min and extension at 72 °C for 1 min; and a final extension at 72 °C for 3 min.

Amplicons were analyzed by electrophoresis on a 1.0% agarose gel stained with ethidium bromide (2 μL/mL) (Sigma-Aldrich, St. Louis, MO, USA) under standard conditions. DNA bands of approximately 800 nucleotides were purified using the NucleoSpin^®^ Gel and PCR Clean-up Kit (Macherey-Nagel, Düren, Germany).

PCR products derived from dogs 1–7 was sent for bidirectional Sanger sequencing to Eurofins Scientific (Luxembourg). The resulting sequences were submitted to GenBank under accession numbers PX132602 (dog 1), PX132603 (dog 2), PX132604 (dog 3), PX132598 (dog 4), PX132599 (dog 5), PX132600 (dog 6), and PX132601 (dog 7).

Multiple sequence alignments of the obtained *gC gene* sequences were performed with *gC gene* sequences retrieved from GenBank. Phylogenetic analysis was subsequently conducted using EMBL-EBI Clustal Omega (1.2.4) (Hinxton, Cambridgeshire, UK) bioinformatic tool, employing the appropriate algorithm and evolutionary model.

## 3. Results

### 3.1. Histopathological Findings

**Figure 3:**
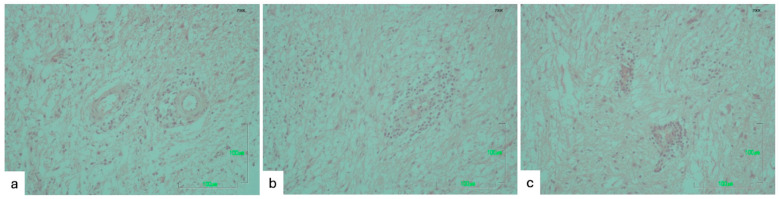
Non-suppurative encephalitis (consisted of mild perivascular cuffing of mononuclear cells) in the brainstem of the three euthanized crossbreed hunting dogs from Thessaly region, November 2024 ((**a**–**c**), from dogs 6, 7, and 8, respectively), H-E stain, 200×, Bar 100 μm.

No specific histopathological lesions, except a mild vascular congestion were observed in the cerebrum and cerebellum microsections. However, a non-suppurative encephalitis (Figure 3) and a mild glial proliferation were observed in the brainstem microsections, as they have already been described elsewhere [36].

### 3.2. Laboratory Detection of PRV by PCR

All brain tissue homogenates collected from the examined dogs tested positive for PRV using PCR. The molecular detection of PRV confirmed the initial clinical diagnosis made by the attending veterinarians. The observed neurological symptoms, particularly the intense pruritus localized on the head combined with the fact that all dogs were used for hunting and had recent direct contact with feral swine, strongly supported the hypothesis of PRV infection. This hypothesis was conclusively verified by the molecular analysis of the samples.

### 3.3. Sequence Comparison and Phylogenetic Analysis

To genetically characterize the PRV isolates obtained from dogs 1 tο 7, partial nucleotide sequences of the *gC gene* were generated through Sanger sequencing of PCR products. Unfortunately, the poor quality Sanger sequencing for dog 8 isolate did not allow its inclusion in the analysis. Sequence alignment was performed to assess nucleotide identities among PRV strains isolated from the Thessaly and Epirus regions, and to compare them with two previously reported Greek PRV strains, Hercules (KT983810) and Kolchis (KT983811), isolated from the region of Central Macedonia [37].

The Epirus isolates collected in 2021 and 2024 are closely related to each other and they form a single cluster on the phylogenetic tree, indicating very low genetic divergence and high genetic stability over time. In addition, the two Thessaly strains are also very close related to each other belonging to the same subclade. The data presented in the phylogenetic tree reveal that the Thessaly strains are not related to the Epirus strains and are positioned in a different branch of the tree. When compared with the previously identified Greek strains, the Thessaly lineage was clearly distinct from the Hercules (KT983810) and Kolchis (KT983811) lineage while the Epirus isolates were tightly related with them.

**Figure 4:**
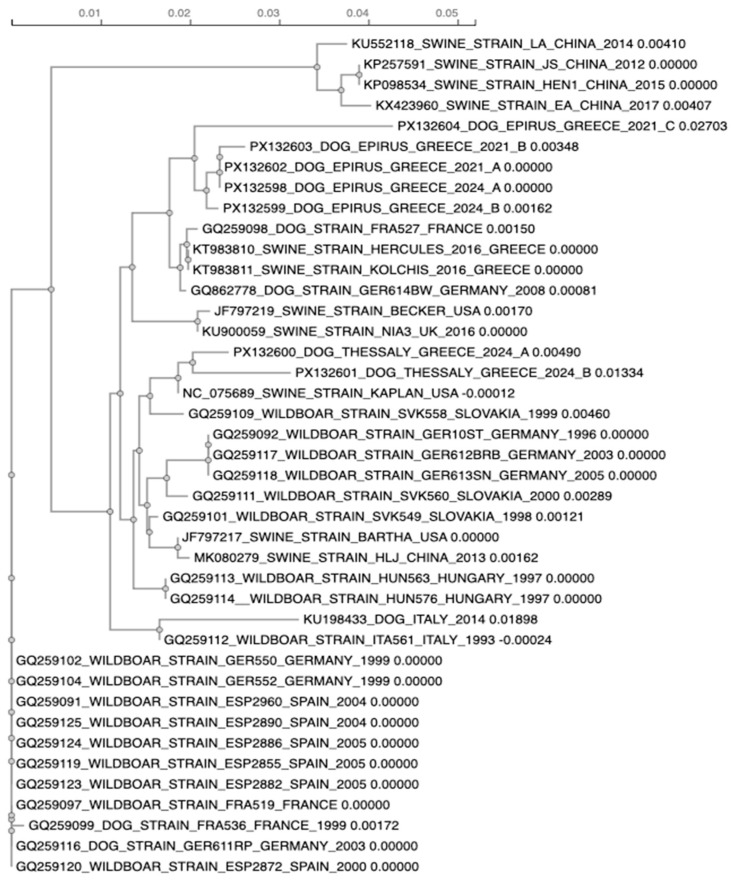
Phylogenetic tree based on partial nucleotide sequences of the *glycoprotein C (gC) gene* from pseudorabies virus (PRV) strains isolated from various geographical regions worldwide. Multiple sequence alignment was performed using CLUSTAL Omega (EMBL-EBI). Numbers following strain names indicate branch lengths, expressed as genetic distances.

The phylogenetic analysis of the *gC gene* sequences from the Greek isolates and their genetic relationship to other PRV strains from Europe, Asia, and America is illustrated in Figure 4.

## 4. Discussion

Feral swine have the broadest geographic distribution of any terrestrial mammal [2]. These widely dispersed populations serve as reservoirs for numerous pathogens transmissible not only to domestic pigs but also to a range of other animal species [14]. Among these pathogens, PRV is of particular concern. Numerous studies across Europe have documented PRV circulation in wild boar populations, with higher seroprevalence observed in Mediterranean countries compared to Central and Northern Europe [16]. In Italy, seroprevalence in wild boars ranges from 7.9% in the northern regions to 23.85% in the southern parts of the country. In Spain, PRV antibodies have been detected in 36.63% of tested wild boars [38], while much lower seroprevalence has been recorded in Switzerland, the Netherlands, and Sweden—up to 4% [39,40,41,42]. In Greece, previous studies reported seroprevalence rates of 32% and 35% in wild boar sera [33,34], with another study identifying PRV DNA in 17.3% of samples from hunted wild boars [35].

Dogs are among the animal species that can frequently come into close contact with wild boars, particularly during hunting. Although PRV is endemic in many wild boar populations, awareness of the infection risk among hunting dog owners remains limited. Infection in dogs typically occurs through direct or indirect contact with infected wild boars, as dogs are considered dead-end hosts and do not shed the virus in quantities sufficient to infect other dogs [31]. In Greece, as in many other countries, dogs are commonly used for tracking wild boars during hunts indicating their location to the hunter. This activity often brings them into direct physical contact with the hunted animal, increasing their exposure to PRV and other pathogens carried by wild swine [14]. Moreover, it is a common—though hazardous—practice among hunters to feed raw offal or meat from hunted boars to their dogs, which constitutes an additional route of infection. Similarly, some pig farmers feed dead piglets to farm dogs, a habit that poses a substantial risk in areas where pseudorabies is endemic, as infected piglets may serve as a source of transmission. Notably, a case has been reported in which a pet dog contracted PRV after consuming raw pork purchased from a butcher shop, with the meat having been imported from Hungary, where PRV was present at the time [43]. The risk extends beyond domestic dogs to include wildlife species such as foxes, wolves, and bears that may share habitats with wild boars or consume their carcasses, potentially impacting biodiversity through the spillover of PRV [20,44].

In the present study, PRV DNA was confirmed in brain tissues from eight hunting dogs in Greece, all of which had reportedly been in direct contact with wild boars. These cases occurred during the national hunting season, which extends from mid-September to the end of February. All dogs had been vaccinated against rabies, and the observed neurological symptoms, in line with laboratory confirmation, pointed toward PRV infection. PCR targeting a fragment of the *gC gene* validated this diagnosis, while necropsy and histopathological findings provided additional support. The *gC gene* of PRV is a preferred target for sequencing and phylogenetic analysis as it combines sufficient genetic variability to discriminate between strains with enough sequence conservation to ensure reliable amplification and alignment. Its involvement in host interaction and immune evasion offers valuable evolutionary insights, while its extensive use in previous studies facilitates robust comparative analyses [29,45,46].

The phylogenetic analysis of the Greek PRV isolates from Epirus and Thessaly revealed the presence of multiple, genetically distinct lineages circulating in different regions and time periods. The two Thessaly 2024 isolates were nearly identical to each other and clustered with the strain Kaplan. This lineage was clearly separated from all Epirus isolates and the previously reported Greek isolates Hercules and Kolchis, indicating a separate introduction event from Central/Western Europe rather than direct epidemiological links with Epirus or Central Macedonia.

In Epirus, the 2021 and 2024 isolates formed a related clade with minimal divergence over the three-year span. This clade fell within the same phylogenetic group as the French strain FRA527, the German isolate GER614BW and the Greek strains Hercules and Kolchis, indicating a Central/Western European origin or a possible link with the region of Central Macedonia. The high genetic stability of the Epirus isolates suggests either ongoing local circulation of the same lineage or repeated introductions from an external source. The persistence of this lineage in Epirus may reflect the role of a stable reservoir in domestic pigs or wild boar, facilitating sustained transmission.

Collectively, these findings highlight the emergence of genetically distinct PRV subpopulations within Greece, likely shaped by geographical and ecological barriers. This underscores the importance of continued molecular surveillance, while further investigation of a larger number of PRV isolates is required to obtain deeper insights into the epidemiology of the disease.

In conclusion, our results emphasize the substantial risk of PRV infection in hunting dogs exposed to wild boars. The occurrence of neurological signs in rabies-vaccinated dogs following hunting activity should prompt veterinarians to include PRV in the differential diagnosis. Given the lack of effective treatment and the rapid, fatal course of the disease in dogs, euthanasia may represent the most humane option in confirmed cases. Increased awareness and preventive measures—including avoiding feeding dogs raw swine meat—are crucial to mitigate the risk of transmission.

## Data Availability

The original contributions presented in this study are included in the article. Further inquiries can be directed to the corresponding author.
